# Supplementary material for: Mitochondrial Protein Density, Biomass, and Bioenergetics as Predictors for the Efficacy of Glioma Treatments
Source: Int J Mol Sci. 2024 Jun 27;25(13):7038. doi: 10.3390/ijms25137038 (PMC11241254; doi:10.3390/ijms25137038)
Supplement: Supplementary file 1 [file ijms-25-07038-s001.zip › ijms-3038519-supplementary.pdf]

Supplementary Table S1.

Mechanism of action and utilized concentrations of compounds selected for the study

Name	Mechanism of action of target	Final concentration	Target compartment or process
**Rotenone**	Inhibition of Complex I	200 nM	Mitochondrial electron transport chain
**Antimycin A**	Inhibition of Complex III	200 nM	Mitochondrial electron transport chain
**Oligomycin**	Inhibition of Complex V	100 nM	Mitochondrial electron transport chain
**Mdivi-1**	Inhibition of mitochondrial division DRP1 (dynamin-related GTPase) and mitochondrial division Dynamin I (Dnm1)	50 μM	Fusion/fission dynamics
**UK-5099**	Inhibition of mitochondrial pyruvate carrier	20 μM	Oxidative phosphorylation
**Etomoxir****	Inhibition of carnitine palmitoyltransferase 1 (CPT1)	20 μM	Fatty acid oxidation
**BPTES**	Inhibition of glutaminase GLS1	2 μM	Glutaminolysis
**2-Deoxy-D-Glucose (2-DG)***	Inhibition of hexokinase 2 (HK2)	2 mM	Glycolysis
**AZD3965****	Inhibition of monocarboxylate transporter 1	10 nM	Lactate export
**EIPA (Ethylisopropylamiloride)**	Inhibition of sodium-hydrogen exchangers (NHE)	10 μM	pH balance
**Concanamycin A**	Inhibition of vacuolar H-ATPase	2 nM	Endosomes and lysosomes
**Temozolomide***	Alkylation/methylation of guanine residues	20 μM	DNA
**Carmustine***	Alkylation/methylation of guanine residues	20 μM	DNA
**Cyclosporine A* (CsA)**	Binding of cyclophilin D (Cyp D)	1 μM	Permeability transition pore (mPTP)
**Stavudine***	Inhibition of reverse transcriptase (NRTI)	5 μM	mtDNA synthesis
**Dimethyl fumarate***	Modification of cysteine residues, epigenetics	10 μM	TCA cycle, succination

*Clinically approved compounds

**Compounds evaluated in clinical trails

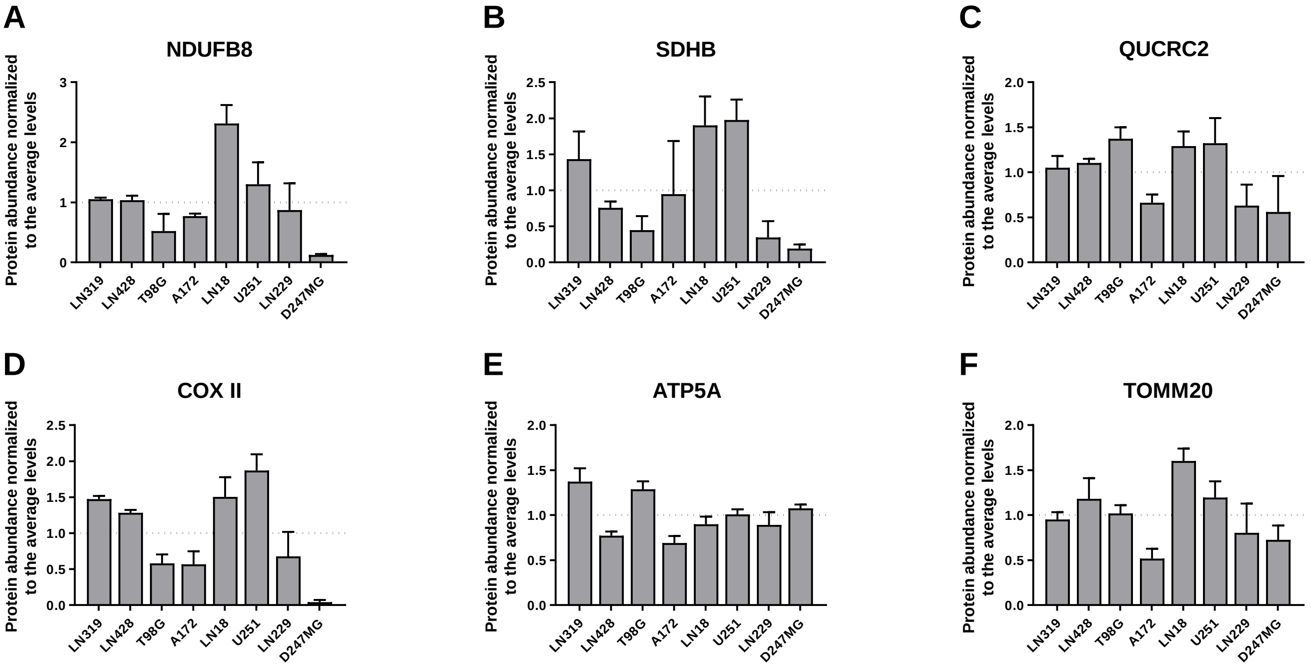

Supplementary Figure S1. A-F C Western blot analysis of 6 mitochondrial proteins in lysates of glioma cells. The abundance of proteins was normalized to the mean levels. Values are means ± SEM, n = 3 (biological replicates).

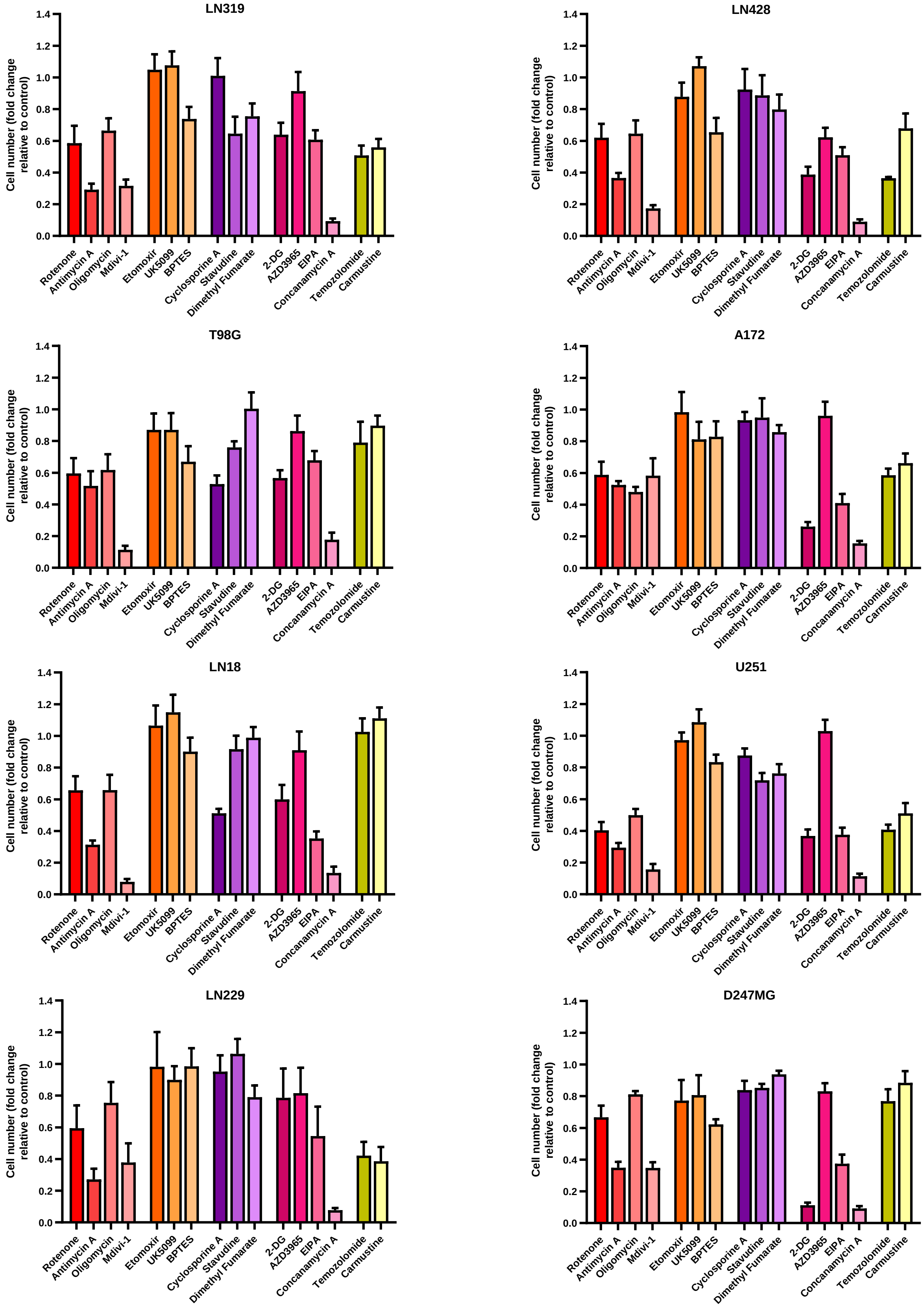

Supplementary Figure S2. Glioma cells were seeded one day prior to the beginning of the experiment and then treated with selected drugs for 72 h. Subsequently, analysis of cell number was carried out. The changes in cell number were quantified using flow cytometry. Only events corresponding to normal cell size were considered. Values are means ± SEM, n = 3-4 (biological replicates).

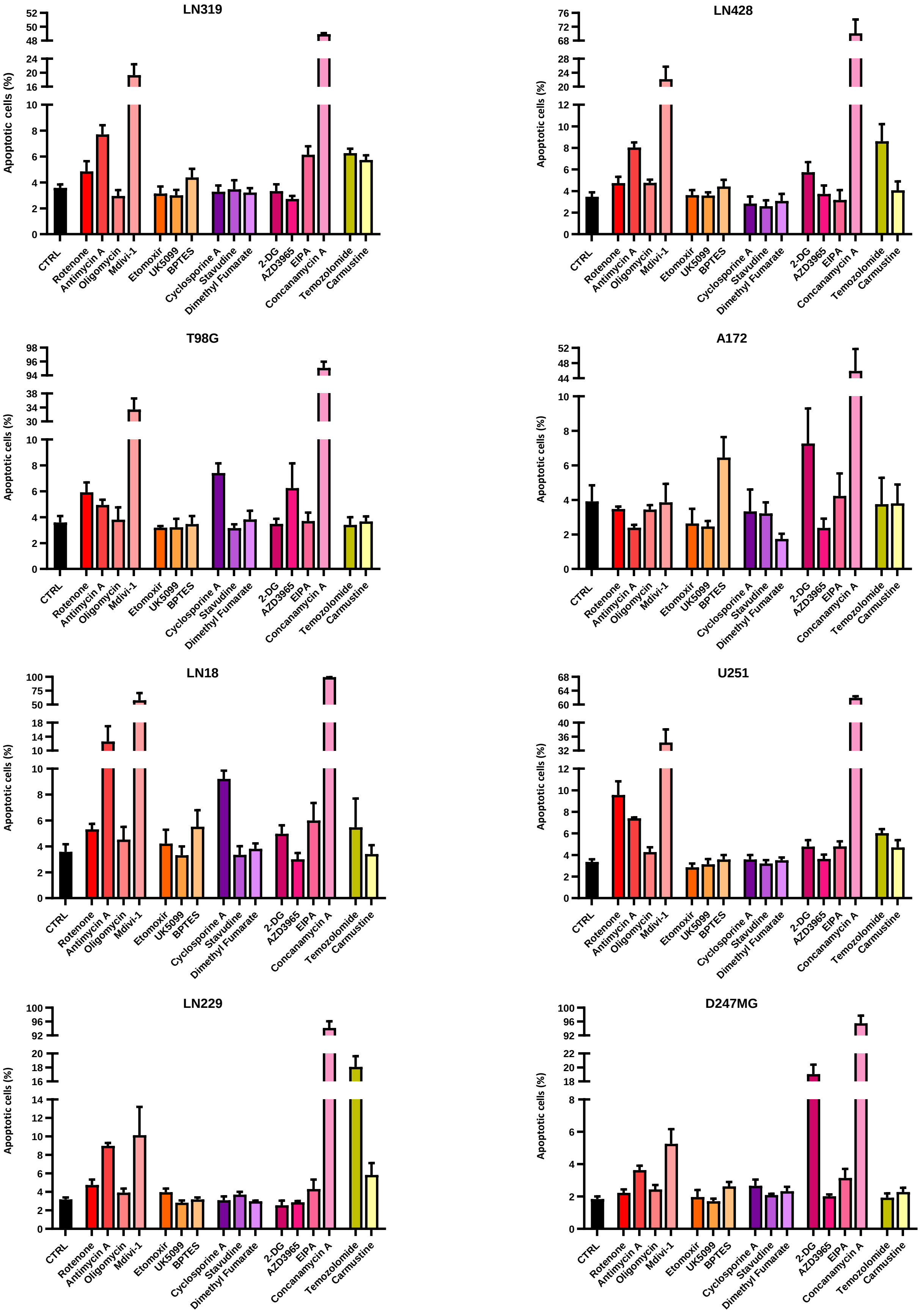

Supplementary Figure S3. Glioma cells were seeded one day prior to the beginning of the experiment and then treated with selected drugs for 72 h. Subsequently, analysis of apoptosis levels was carried out. The changes in apoptosis levels were quantified using flow cytometry along with annexin V staining. Values are means ± SEM, n = 3-4 (biological replicates).

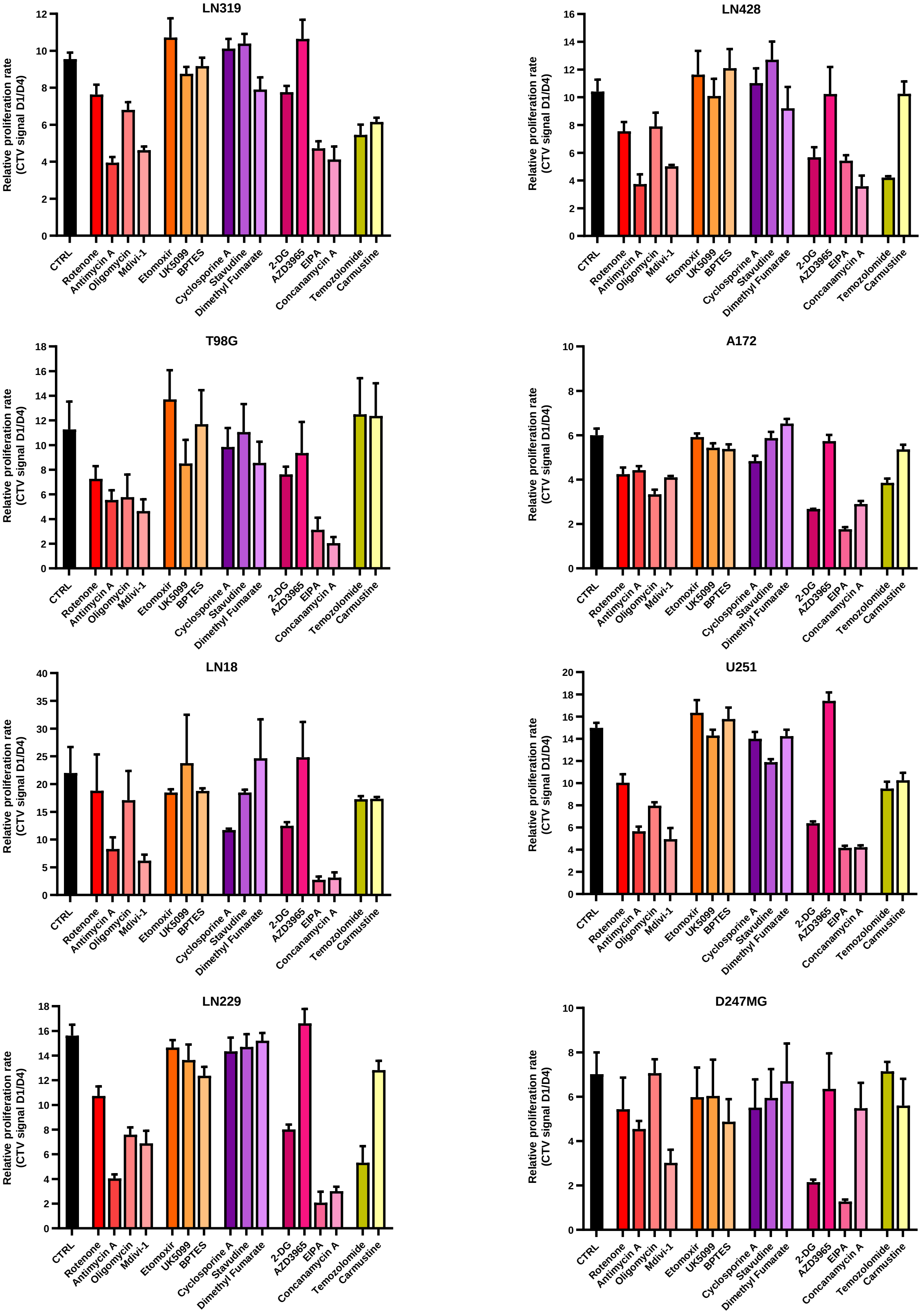

Supplementary Figure S4. Glioma cells were prestained with 3 μM of CellTrace Violet dye and seeded one day prior to the start of the experiment. On day 1 (D1), they were treated with selected drugs for 72 hours. Subsequently, the proliferation rate was analyzed on day 4 (D4). The proliferation rate was calculated using the D1/D4 ratio. Values are means ± SEM, n = 3-4 (biological replicates).

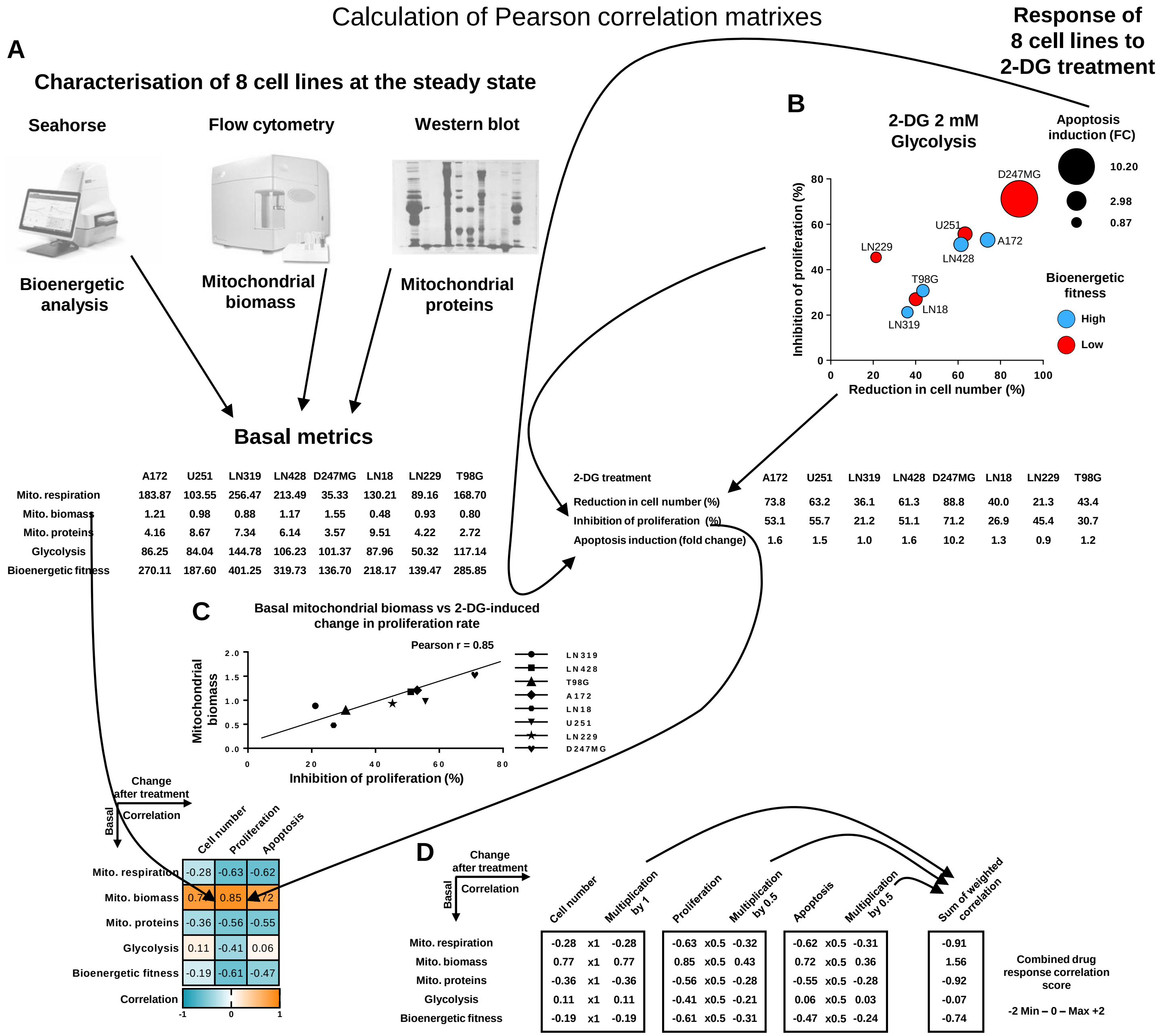

Supplementary Scheme S1. Glioma cells were either analysed at the steady state or following the 72 h treatment with 2-DG. **A** Bioenergetic characteristics and mitochondrial parameters were obtained at the steady state using seahorse assay, flow cytometry and western blotting. **B** Following the 72 h 2-DG treatment, the analysis of cell number, apoptosis levels and proliferation rate, was carried out. The obtained data was quantified and represented as fold changes or percentages over the control condition. Bubble plots illustrate the mean changes in cell number (x-axis, %), mean changes in proliferation rate (y-axis, %), and mean changes in apoptosis (size, fold change). Values are means, n = 3-4 (biological replicates). The color-coding indicates whether a cell line displayed increased bioenergetic activity (blue) or decreased (red) relative to the average level. The changes in cell number, proliferation rate, and apoptosis level were quantified using flow cytometry along with annexin V or CellTrace Violet assays. Following the evaluation of steady state-characteristics and cell response to 2-DG treatment we assessed the linkage between these parameters. **C (top)** Illustration of strong positive correlations between mitochondrial biomass and inhibition of proliferation induced by 2-DG. **C (bottom)** Correlation matrix depicting the relationships between various mitochondrial and bioenergetic characteristics measured at the steady level and the changes in cell number, proliferation and apoptosis rates induced by 2-DG. **D** To better understand the relationship between mitochondrial/bioenergetic characteristics and cell responses to drug treatments, we introduced a "combined drug response correlation score." This score integrates the treatment-induced changes in cell number, proliferation rate, and apoptosis levels, with weighted values to reflect their significance. Specifically, the change in cell number is given a weight of 1.0, acknowledging its primary importance, while changes in proliferation and apoptosis are each weighted at 0.5. Thus, the score is calculated as the sum of these weighted correlation coefficients, represented as 1.0 x P_{CCvsBM} (treatment-induced change in cell number vs basal metric) + 0.5 x P_{PCvsBM} (treatment-induced change in proliferation rate vs basal metric) + 0.5 x P_{ACvsBM} (treatment-induced change in apoptosis levels vs basal metric), where P represents Pearson correlation coefficients. This provides a broader assessment of drug effects, ranging from -2 to 2, thereby offering a wider range than the conventional Pearson coefficient scale of -1 to 1.

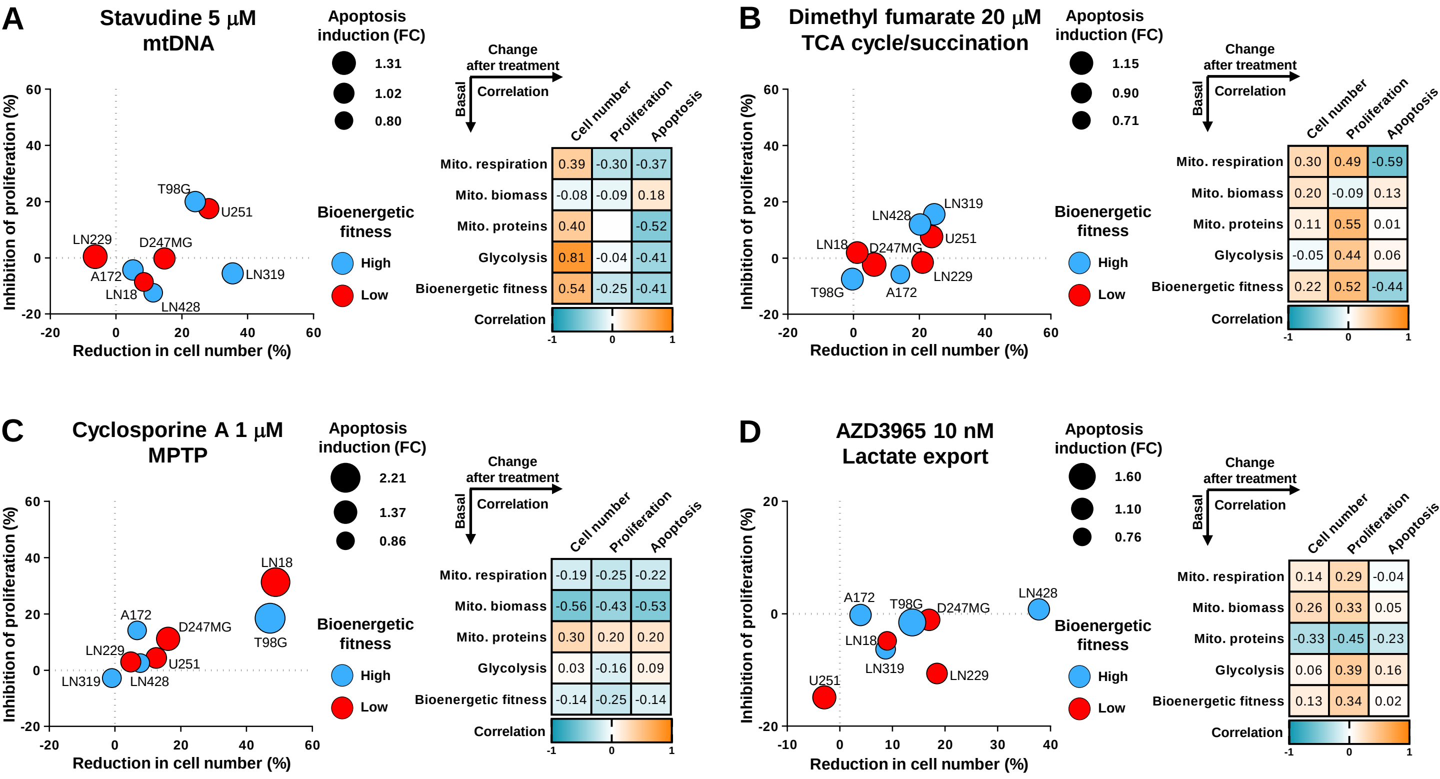

Supplementary Figure S5. A-D Glioma cells were seeded one day prior to the beginning of the experiment and then treated with selected drugs in indicated concentrations for 72 h. Subsequently, analysis of cell number, apoptosis levels and proliferation rate, was carried out (Figures S2-S4). The obtained data was quantified and represented as fold changes or percentages over the control condition. Values are means, n = 3-4 (biological replicates). **A-D Left side:** Bubble plots illustrate the mean changes in cell number (x-axis, %), mean changes in proliferation rate (y-axis,%), and mean changes in apoptosis (size, fold change). The color-coding indicates whether a cell line displayed increased bioenergetic activity (blue) or decreased (red) relative to the average level. The changes in cell number, proliferation rate, and apoptosis level were quantified using flow cytometry along with annexin V or CellTrace Violet assays. **A-D Right side:** Correlation matrices depict the relationships between various mitochondrial and bioenergetic characteristics measured at the steady level and the drug responses induced by treatments with selected compounds. The drug response is represented by changes in the three parameters of cell number, proliferation rate, and apoptosis.

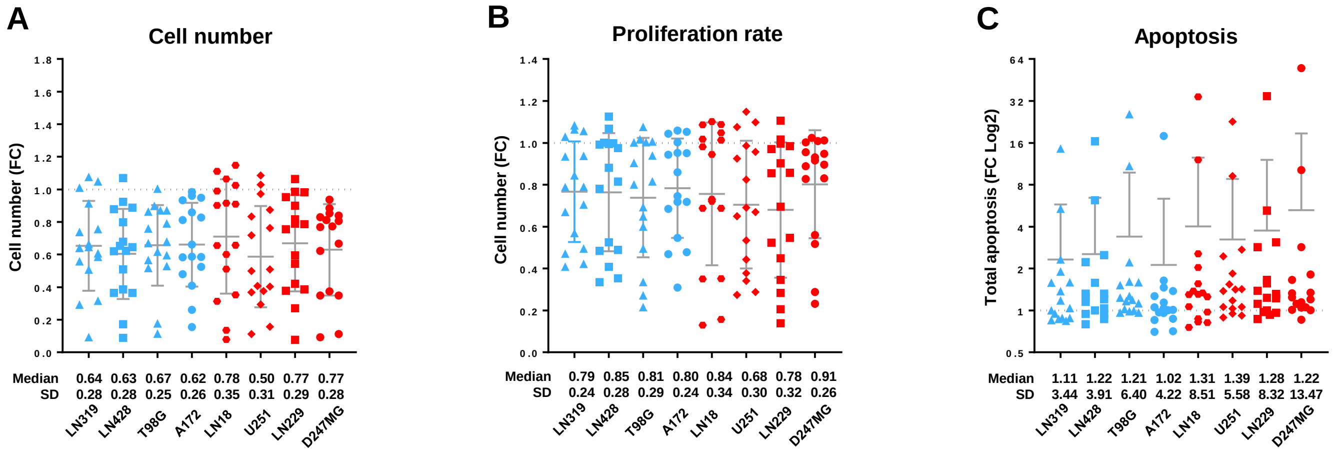

Supplementary Figure S6. A-C Summary plots depicting the changes in cell number, apoptosis, proliferation rate, upon treatment of cells with 16 different compounds. The color-coding indicates whether a cell line displayed increased bioenergetic activity (blue) or decreased (red) relative to the average level. The graphs represent a summary and contain data derived from other figures. Each dot represents the mean effect of one compound, n = 3-4 (biological replicates). The lines depict the mean effect of 16 drugs, error bars are ± SD.
